# 2020 JAPAN Critical Limb Ischemia Database (JCLIMB) Annual Report

**DOI:** 10.3400/avd.ar.23-00096

**Published:** 2024-02-05

**Authors:** The Japanese Society for Vascular Surgery JCLIMB Committee, NCD JCLIMB Analytical Team

**Keywords:** arterial occlusive disease, leg ischemia, peripheral arterial disease (PAD), CLI, annual report

## Abstract

Since 2013, the Japanese Society for Vascular Surgery has started the project of nationwide registration and tracking database for patients with critical limb ischemia (CLI) who are treated by vascular surgeons. The purpose of this project is to clarify the current status of the medical practice for the patients with CLI to contribute to the improvement of the quality of medical care. This database, called JAPAN Critical Limb Ischemia Database (JCLIMB), is created on the National Clinical Database and collects data of patients’ background, therapeutic measures, early results, and long-term prognosis as long as 5 years after the initial treatment. The limbs managed conservatively are also registered in JCLIMB, together with those treated by surgery and/or endovascular treatment. In 2020, 1299 CLI limbs (male 890 limbs: 69%) were registered by 85 facilities. Arteriosclerosis obliterans has accounted for 99% of the pathogenesis of these limbs. In this manuscript, the background data and the early prognosis of the registered limbs are reported. (This is a translation of Jpn J Vasc Surg 2023; 32: 363–391.)

## 1. Introduction

Recently, an increasing number of patients with critical limb ischemia (CLI) are undergoing medical care at clinical practice sites. Improving the outcome of treatment for these patients is an important and urgent issue. Since 2013, the Japanese Society for Vascular Surgery (JSVS) has initiated the project of a nationwide CLI registration and tracking database to obtain CLI epidemiological data that can be shared among the medical staff. The background of CLI limbs, contents of treatment, early outcome, and long-term outcome until 5 years after surgery, including non-surgical limbs, are registered in this database. The database was named JAPAN Critical Limb Ischemia Database (JCLIMB) and established on the National Clinical Database (NCD). The JCLIMB project’s primary objective is to clarify the current status of CLI treatment performed by vascular surgeons in Japan and inform physicians at practice sites, thus improving the quality of medical care. The initial registration data, and their tracking data 1 month after registration in 2013–2019, have already been published.^1^^–^^7^^)^ This article reports the basic data registered in 2020.

## 2. JCLIMB

Registration details, including the definition of CLI, have already been described in the 2013 annual report.^1)^ CLI to be registered was defined according to Trans-Atlantic Inter-Society Consensus Document on Management of Peripheral Arterial Disease (TASC II)^8)^: chronic ischemic rest pain, ulcers, or gangrene attributable to objectively proven arterial occlusive disease. CLI diagnosis should be confirmed by ankle pressure (AP) below 50 mmHg or by toe pressure (TP) below 30 mmHg in limbs with rest pain, and done by AP below 70 mmHg or by TP below 50 mmHg in limbs with ulcer or gangrene.^8)^

The same limb can be registered in the JCLIMB only once within a 5-year tracking period. When the registered limb is treated at different times or at different institutions, such data should be added only to the tracking items of each limb in the JCLIMB, avoiding registration overlap. However, details of the procedure are registered each time in the NCD apart from the registration in the JCLIMB. On the other hand, the patient with bilateral CLI can be registered twice for each limb. Based on NCD regulations, fixing JCLIMB data is done as follows:

Initial registration data: Early April in the following year, tracking data early after treatment (1 month)/6 months after treatment: end of December in the following year, tracking data one year after treatment: end of December after 2 years.

Tracking data 2 years after treatment: end of December after 3 years

Tracking data 3 years after treatment: end of December after 4 years

Tracking data 4 years after treatment: end of December after 5 years

Tracking data 5 years after treatment: end of December after 6 years.

As a general rule, the timing of tracking data registration is accepted within a ±2-month range until 12 months after treatment and within a ±3-month range thereafter. Although the day for tracking data fixing is specified, it is made flexible because, in some limbs, follow-up data might be revealed later.

It is very difficult to require facilities participating in the NCD to register CLI data since a great number of registration items in the JCLIMB would put too much burden on them. Thus, facilities wishing to participate were recruited. In total, 85 facilities, which registered CLI limbs in 2020 at the time of compiling in December 2022, are listed in the appendix.

Since the JCLIMB is positioned as a registry study on the NCD, patient consent to participate in the study and the ethical review of the study at the time of participation in the NCD were adopted.

## 3. Comments on the Aggregated Data in 2020

The initial registration data in 2020 were fixed in early April 2021, and the tracking data early after treatment (1 month) were fixed in April 2022. In December 2022, 1299 limbs, those of 890 males (69%) and 409 females (31%), were registered in 85 facilities. All data and extracted data on arteriosclerosis obliterans (ASO) were collected according to the registered items. Since ASO accounted for 99% of all limbs, the overall and ASO data showed similar tendencies. In the comments, ASO data were presented in parentheses. In addition, because the Society for Vascular Surgery (SVS)’s WIfI classification was reported in 2014 (**Tables 1-1-1–1-1-3**),^9)^ JCLIMB made several changes and additions to the registered items, making the WIfI classification possible since 2015 (**Tables 1-2-1–1-2-3**). The total figure was not always consistent, mostly due to missing values, and an explanation for each inconsistency was added. Although, in July 2019, the registration format for bilateral simultaneous surgery was changed from 2 limbs in 2 patients to 2 limbs in 1 patient in the NCD, the data were calculated on a limb basis as before in the 2019 JCLIMB annual report in order to eliminate the discrepancy with the past reports.

**Table:** Table 1-1 SVS WIfI classification original^9)^ Table 1-1-1 Wound

Grade	Ulcer	Gangrene
0	No ulcer	No gangrene
	Clinical description: ischemic rest pain (requires typical symptoms + ischemia grade 3), no wound	
1	Small, shallow ulcer(s) on distal leg or foot; no exposed bone, unless limited to distal phalanx	No gangrene
	Clinical description: minor tissue loss. Salvageable with simple digital amputation (1 or 2 digits) or skin coverage	
2	Deeper ulcer with exposed bone, joint or tendon; generally not involving the heel; gangrenous changes limited to digits; shallow heel ulcer, without calcaneal involvement	Gangrenous changes limited to digits
	Clinical description: major tissue loss salvageable with multiple (3) digital amputations or standard TMA ± skin coverage	
3	Extensive, deep ulcer involving forefoot and/or midfoot; deep, full thickness heel ulcer ± calcaneal involvement	Extensive gangrene involving forefoot and/or midfoot; full thickness heel necrosis 6 calcaneal involvement
	Clinical description: extensive tissue loss salvageable only with a complex foot reconstruction or nontraditional TMA (Chopart or Lisfranc); flap coverage or complex wound management needed for large soft tissue defect	

SVS: Society for Vascular Surgery; TMA: transmetatarsal amputation

**Table:** Table 1-1-2 Ischemia

Grade	ABI	AP (mmHg)	TP, TcPO_2_ (mmHg)
0	≥0.80	>100	>60
1	0.60–0.79	70–100	40–59
2	0.40–0.59	50–70	30–39
3	≤0.39	<50	<30

Patients with diabetes should have TP measurements. If arterial calcification precludes reliable ABI or TP measurements, ischemia should be documented by TcPO_2_, SPP, and PVR. If TP and ABI measurements result in different grades, TP will be the primary determinant of ischemia grade.

Flat or minimally pulsatile forefoot PVR = grade 3.

ABI: Ankle brachial (pressure) index; AP: ankle pressure; PVR: pulse volume recording; SPP: skin perfusion pressure; TP: toe pressure; TcPO_2:_ transcutaneous oximetry

**Table:** Table 1-1-3 Foot infection

Grade	Clinical manifestation of infection	IDSA/PEDISInfection severity*
0	No symptoms or signs of infection	Uninfected
1	Infection present, as defined by the presence of at least 2 of the following items: Mild • Local swelling or induration • Erythema >0.5 to 2 cm around the ulcer • Local tenderness or pain • Local warmth Purulent discharge (thick, opaque to white, or sanguineous secretion) Local infection involving only the skin and the subcutaneous tissue (without involvement of deeper tissues and without systemic signs as described below). Exclude other causes of an inflammatory response of the skin (e.g., trauma, gout, acute Charcot neuro-osteoarthropathy, fracture, thrombosis, venous stasis)	Mild
2	Local infection (as described above) with erythema >2 cm or involving structures deeper than skin and subcutaneous tissues (e.g., abscess, osteomyelitis, septic arthritis, fasciitis), and no systemic inflammatory response signs (as described below)	Moderate
3	Local infection (as described above) with the signs of SIRS, as manifested by two or more of the following: Temperature >38°C or <36°C • Heart rate >90 beats/min • Respiratory rate >20 breaths/min or PaCO_2_ <32 mmHg • White blood cell count >12000 or <4000 cu/mm or 10% immature (band) forms	Severe

An ischemia may complicate and increase the severity of any infection. Systemic infection may sometimes manifest with other clinical findings, such as hypotension, confusion, vomiting, or evidence of metabolic disturbances, such as acidosis, severe hyperglycemia, and new-onset azotemia.

*SVS adaptation of IDSA and IWGDF perfusion, extent/size, depth/tissue loss, sensation (PEDIS) classifications of diabetic foot infection.

PACO_2_: partial pressure of arterial carbon dioxide; SIRS: systemic inflammatory response syndrome; SVS: Society for Vascular Surgery; IDSA: Infectious Diseases Society of America; IWGDF: International Working Group on the Diabetic Foot

**Table:** Table 1-2 SVS WIfI classification: correlation of WIfI and items in JCLIMB Table 1-2-1 Wound

Grade	Rutherford classification	Ulcer		Sites of gangrene
		Depth of ulcer (University of Texas classification: grade)	Sites of ulcer	
0	Class 4		No ulcer	No gangrene
1	Class 5, 6	I II, III	Any portion Limited to digits	No gangrene
2	Class 5, 6	I II, III	Heel Foot: distal metatarsal excluding heel	Limited to digits
3	Class 5, 6	II, III	Foot: proximal metatarsal, heel, ankle, lower leg	Extensive proximal to fore foot

SVS: Society for Vascular Surgery; JCLIMB: JAPAN Critical Limb Ischemia Database

**Table:** Table 1-2-2 Ischemia^21)^

Grade	SPP
0	≥50 mmHg
1	40–49 mmHg
2	30–39 mmHg
3	<30 mmHg

SPP: skin perfusion pressure

**Table:** Table 1-2-3 Foot Infection

Grade	Local infection, foot	Systemic infection (SIRS)
0	(−)	(−)
1	(+) Involving only the skin and the subcutaneous tissue (erythema around the ulcer; 0.5–2 cm)	(−)
2	(+) Involving only the skin and the subcutaneous tissue (erythema around the ulcer; >2 cm) or involving structures deeper than skin and subcutaneous tissues (e.g., abscess, osteomyelitis, septic arthritis, fasciitis)	(−)
3	(+)	(+)

SIRS: systemic inflammatory response syndrome

### (1) Pretreatment patients’ background

Pretreatment patients’ background is shown in **Tables 2-1–2-6**. Good blood pressure control was defined as below 140/90 mmHg, without diabetes and renal failure, or below 130/80 mmHg with these diseases. Diabetes control was considered good when hemoglobin A1c was below 7.0% (national glycohemoglobin standardization program [NGSP] value). Dyslipidemia control was considered good when low-density lipoprotein was below 100 and 80 mg/dL in the absence and presence of other arteriosclerotic diseases, respectively. The presence of heart failure was judged clinically. The patient was regarded as having heart failure based on a past history of admission due to heart failure, clinical symptoms of heart failure, a diagnosis of heart failure confirmed by echocardiography, or reduced cardiac function on echocardiography even with no clinical heart failure symptoms. Renal dysfunction was graded following the new chronic kidney disease severity classification of the “Clinical Practice Guidebook for Diagnosis and Treatment of Chronic Kidney Disease 2012”^10)^: Renal dysfunction was absent when the estimated glomerular filtration rate (eGFR) (mL/min/1.73 m^2^) was 60 or higher, and it was graded as G3a, G3b, G4, and G5 when eGFR was 45–59, 30–44, 15–29, and below 15, respectively. eGFR below 15 in hemodialysis patients was graded as G5D.

**Table:** Table 2 Patients’ background Table 2-1 Patients’ background 1

a. Total																		
	n	Sex		Laterality		BMI (Median)	Pathogenesis				Age at registration							
		Male	Female	Right	Left		ASO	TAO	Vasculitis	others	ASO		TAO		Vasculitis		Others	
											Mean (±SD)		Mean (±SD)		Mean (±SD)		Mean (±SD)	
Rutherford 4	304	200	104	163	141	21.7	300	0	0	4	75.4	(9.4)	–	–	–	–	75.5	(15.6)
Rutherford 5	807	561	246	390	417	21.5	793	2	10	2	74.7	(10.1)	60.5	(6.5)	71.1	(11.4)	73.5	(0.5)
Rutherford 6	188	129	59	98	90	21.0	187	0	1	0	73.7	(11.6)	–	–	81.0	(0.0)	–	–
Total	1299	890	409	651	648	21.5	1280	2	11	6	74.7	(10.1)	60.5	(6.5)	71.8	(10.8)	75.3	(11.9)
b. ASO																		
	n	Sex		Laterality		BMI (Median)	Age at registration											
		Male	Female	Right	Left		Mean (±SD)											
Rutherford 4	300	197	103	161	139	21.7	75.4	(9.4)										
Rutherford 5	793	554	239	379	414	21.4	74.7	(10.1)										
Rutherford 6	187	129	58	98	89	21.1	73.7	(11.6)										
Total	1280	880	400	638	642	21.5	74.7	(10.1)										

Vasculitis: Takayasu's arteritis, Collagen disease, Behcet disease, FMD, etc., excluding TAO.

Others: others including debranch bypasses for TEVAR or EVAR.

Simultaneous bilateral treatments in one case were counted as 2 limbs in 2 cases.

ASO: arteriosclerosis obliterans; TAO: thromboangiitis obliterans; SD: standard deviation; FMD: fibromuscular dysplasia; BMI: body mass index; TEVAR: thoracic endovascular aortic aneurysm repair; EVAR: endovascular aortic aneurysm repair

**Table:** Table 2-2 Patients’ background 2

a. Total															
	Diabetes			Diabetes therapy			Hypertension			Dyslipidemia			Smoking		
	(−)	(+)		Diet therapy	Medication	Insulin therapy	(−)	(+)		(−)	(+)		(−)	(+)	
		Management						Management			Management			Ex-smoker	Current smoker
		Good	Poor					Good	Poor		Good	Poor			
Rutherford 4	133	148	23	17	102	52	66	219	19	142	149	13	131	125	48
Rutherford 5	273	406	128	72	260	202	184	564	59	430	341	36	309	377	121
Rutherford 6	60	79	49	13	70	45	40	127	21	98	79	11	66	101	21
Total	466	633	200	102	432	299	290	910	99	670	569	60	506	603	190
b. ASO															
	Diabetes			Diabetes therapy			Hypertension			Dyslipidemia			Smoking		
	(−)	(+)		Diet therapy	Medication	Insulin therapy	(−)	(+)		(−)	(+)		(−)	(+)	
		Management						Management			Management			Ex-smoker	Current smoker
		Good	Poor					Good	Poor		Good	Poor			
Rutherford 4	132	145	23	17	99	52	65	217	18	141	146	13	129	124	47
Rutherford 5	264	403	126	72	257	200	179	557	57	422	336	35	301	373	119
Rutherford 6	59	79	49	13	70	45	40	126	21	98	78	11	65	101	21
Total	455	627	198	102	426	297	284	900	96	661	560	59	495	598	187

Blood pressure management good: diabetes or renal failure (−) <140/90 mmHg (+) <130/80 mmHg. Diabetes management good: HbA1c <7.0% (NGSP).

Dyslipidemia management good: other sclerotic lesions (−) LDL <100 mg/dL, (+) LDL <80 mg/dL.

ASO: arteriosclerosis obliterans; HbA1c: hemoglobin A1c; LDL: low-density lipoprotein; NGSP: National Glycohemoglobin Standardization Program

**Table:** Table 2-3 Patients' background 3

a. Total														
	Ischemic heart disease				Heart failure		Cerebrovascular disease		Renal dysfunction					
	(−)	(+)			(−)	(+)	(−)	(+)	(−)	(+)				
		Medical treatment	PCI	CABG						G3a	G3b	G4	G5	G5D
Rutherford 4	212	17	43	32	263	41	245	59	114	34	30	14	4	108
Rutherford 5	459	87	173	88	693	114	618	189	220	96	73	36	10	372
Rutherford 6	114	13	43	18	157	31	156	32	60	20	21	10	0	77
Total	785	117	259	138	1113	186	1019	280	394	150	124	60	14	557
b. ASO														
	Ischemic heart disease				Heart failure		Cerebrovascular disease		Renal dysfunction					
	(−)	(+)			(−)	(+)	(−)	(+)	(−)	(+)				
		Medical treatment	PCI	CABG						G3a	G3b	G4	G5	G5D
Rutherford 4	210	17	41	32	259	41	242	58	112	34	30	14	3	107
Rutherford 5	450	85	172	86	682	111	607	186	213	93	71	36	10	370
Rutherford 6	114	12	43	18	156	31	155	32	60	19	21	10	0	77
Total	774	114	256	136	1097	183	1004	276	385	146	122	60	13	554

Heart failure (+): history of admission due to heart failure, clinical symptoms due to heart failure confirmed by ultrasound examination, and apparently decreased cardiac function by ultrasound examination without clinical symptoms.

Renal dysfunction: (−) (60≤), G3a (45–59), G3b (30–44), G4 (15–29), G5 (<15), G5D (<15 with hemodialysis). New CKD risk stratification by eGFR (mL/min/1.73 m2) in “Clinical Practice Guidebook for Diagnosis and Treatment of Chronic Kidney Disease 2012”.

ASO: arteriosclerosis obliterans; PCI: percutaneous coronary intervention; CABG: coronary arterial bypass grafting; eGFR: estimated glomerular filtration rate; CKD: chronic kidney disease

**Table:** Table 2-4 Patients’ background 4

a. Total															
	Malignant neoplasm				Site of malignant neoplasm										
	(−)	(+)			Head and neck	Esophagus	Lung	Stomach	Hepatobiliary pancreas	Colon	Breast	Uterus	Ovarium	Prostate	Others
		History of cancer	Under treatment*	Unknown											
Rutherford 4	286	10	6	2	1	1	2	4	3	1	0	1	2	1	3
Rutherford 5	712	68	24	3	2	1	17	22	2	15	4	2	2	7	23
Rutherford 6	173	8	7	0	1	0	2	4	1	4	1	0	0	2	3
Total	1171	86	37	5	4	2	21	30	6	20	5	3	4	10	29
b. ASO															
	Malignant neoplasm				Site of malignant neoplasm										
	(−)	(+)			Head and neck	Esophagus	Lung	Stomach	Hepatobiliary pancreas	Colon	Breast	Uterus	Ovarium	Prostate	Others
		History of cancer	Under treatment*	Unknown											
Rutherford 4	282	10	6	2	1	1	2	4	3	1	0	1	2	1	3
Rutherford 5	701	65	24	3	2	1	17	21	2	15	3	2	2	7	22
Rutherford 6	172	8	7	0	1	0	2	4	1	4	1	0	0	2	3
Total	1155	83	37	5	4	2	21	29	6	20	4	3	4	10	28

*Including palliative therapy or recurrence

ASO: arteriosclerosis obliterans

**Table:** Table 2-5 Patients' background 5

a. Total													
	Contralateral limb occlusive lesions							Vascular lesions excluding occlusion					
	(−)	(+)						(−)	TAA	AAA (including IAA)	Peripheral artery aneurysm	Carotid stenosis	Others
		Asymptomatic	Intermittent claudication	CLI			Posttreatment						
				R4	R5	R6							
Rutherford 4								272	1	14	2	9	6
Rutherford 5	Not aggregated from 2020 to 2023							728	3	17	2	44	13
Rutherford 6								168	1	6	1	8	4
Total								1168	5	37	5	61	23
b. ASO													
	Contralateral limb occlusive lesions							Vascular lesions excluding occlusion					
	(−)	(+)						(−)	TAA	AAA (including IAA)	Peripheral artery aneurysm	Carotid stenosis	Others
		Asymptomatic	Intermittent claudication	CLI			Posttreatment						
				R4	R5	R6							
Rutherford 4								269	1	13	2	9	6
Rutherford 5	Not aggregated from 2020 to 2023							718	3	16	2	42	12
Rutherford 6								168	1	6	1	8	3
Total								1155	5	35	5	59	21

ASO: arteriosclerosis obliterans; CLI: critical limb ischemia; TAA: thoracic aortic aneurysm; AAA: abdominal aortic aneurysm; IAA: iliac artery aneurysm

**Table:** Table 2-6 Patients' background 6

a. Total								
	Fatty acid							
	Arachidonic acid(AA)		Eicosapentaenoic acid(EPA)		Docosahexaenoic acid(DHA)		EPA/AA	
	n	Median	n	Median	n	Median	n	Median
Rutherford 4	10	139.6	10	47.0	10	122.5	10	0.3
Rutherford 5	36	159.2	36	50.3	36	100.4	36	0.3
Rutherford 6	7	158.5	7	33.2	7	95.6	7	0.2
Total	53	157.0	53	47.0	53	99.8	53	0.3
b. ASO								
	Fatty acid							
	Arachidonic acid(AA)		Eicosapentaenoic acid(EPA)		Docosahexaenoic acid(DHA)		EPA/AA	
	n	Median	n	Median	n	Median	n	Median
Rutherford 4	10	139.6	10	47.0	10	122.5	10	0.3
Rutherford 5	36	159.2	36	50.3	36	100.4	36	0.3
Rutherford 6	7	158.5	7	33.2	7	95.6	7	0.2
Total	53	157.0	53	47.0	53	99.8	53	0.3

ASO: arteriosclerosis obliterans; AA: arachidonic acid; EPA: Eicosapentaenoic acid; DHA: docosahexaenoic acid

The causes of the arterial occlusion of the limb were ASO in 1280 (99%) limbs, thromboangiitis obliterans (TAO) in 2, vasculitis (Takayasu’s arteritis, collagen disease, Behçet’s disease, and fibromuscular dysplasia excluding TAO) in 11, and others in 6. Comorbidities consisted of diabetes in 64% (64%) of the limbs, hypertension in 78% (78%), dyslipidemia in 48% (48%), ischemic heart disease in 40% (40%), heart failure in 14% (14%), cerebrovascular disease in 22% (22%), dialysis for renal failure in 43% (43%), past medical history of malignant neoplasm or that being treated in 10% (10%), and smoking (ex- and current) in 61% (61%).

The problems and considerations on these spreadsheets are described below. In **Table 2-4**, the total number of malignant neoplasm sites is larger than that of malignant neoplasms. This is because multiple selections of malignant neoplasm sites were possible. The blood flow data (ankle brachial index [ABI], the toe brachial index [TBI], skin perfusion pressure [SPP]) of contralateral limbs were omitted in this report because there were many missing values though they had been displayed until 2018. In addition, aggregation of arterial occlusive lesions of contralateral limbs were given up in 2020 because a setting error in the data input method of arterial occlusive lesions of contralateral limbs made the aggregated numbers unreliable. Although the same situation will continue until 2023, arterial occlusive lesions of contralateral limbs is an important information and will be restored as before from 2024.

### (2) Conditions of limb ischemia

Limb ischemia pretreatment conditions are shown in **Tables 3-1–3-6**. Regarding the walking function (Taylor classification),^11)^ patients who could walk outdoors or indoors independently, including with a cane, were regarded as “ambulatory,” and those unable to walk but able to stand on their own legs during transfer from the bed to a wheel chair were designated as “ambulatory/homebound.”

**Table:** Table 3 Pretreatment condition Table 3-1 Pretreatment condition 1

a. Total																										
	Ambulatory function(Taylor's classification)			Site of ulcer							Depth of ulcer(University of Texas classification: grade)			Site of gangrene							Main site of ulcer/gangrene to be treated					
	Ambulatory	Ambulatory/homebound	Non-ambulatory	Digits	Foot distal metatarsal	Foot proximal metatarsal	Heel	Ankle	Lower leg	Only gangrene w/o ulcer	I	II	III	Digits	Foot distal metatarsal	Foot proximal metatarsal	Heel	Ankle	Lower leg	Only ulcer w/o gangrene	Digits	Foot distal metatarsal	Foot proximal metatarsal	Heel	Ankle	Lower leg
Rutherford 4	218	50	36																							
Rutherford 5	510	175	122	594	77	16	75	16	18	93	468	112	133	373	47	5	24	2	2	385	657	71	14	40	11	14
Rutherford 6	77	55	56	52	44	20	36	5	30	38	40	38	72	73	50	24	32	6	24	23	43	52	27	34	3	29
Total	805	280	214	646	121	36	111	21	48	131	508	150	205	446	97	29	56	8	26	408	700	123	41	74	14	43
b. ASO																										
	Ambulatory function(Taylor's classification)			Site of ulcer							Depth of ulcer(University of Texas classification: grade)			Site of gangrene							Main site of ulcer/gangrene to be treated					
	Ambulatory	Ambulatory/homebound	Non-ambulatory	Digits	Foot distal metatarsal	Foot proximal metatarsal	Heel	Ankle	Lower leg	Only gangrene w/o ulcer	I	II	III	Digits	Foot distal metatarsal	Foot proximal metatarsal	Heel	Ankle	Lower leg	Only ulcer w/o gangrene	Digits	Foot distal metatarsal	Foot proximal metatarsal	Heel	Ankle	Lower leg
Rutherford 4	215	49	36																							
Rutherford 5	502	172	119	585	73	15	74	15	18	92	462	110	128	367	45	5	23	1	2	381	648	68	13	40	10	14
Rutherford 6	77	55	55	51	43	20	36	5	30	38	40	38	71	72	49	24	32	6	24	23	43	51	27	34	3	29
Total	794	276	210	636	116	35	110	20	48	130	502	148	199	439	94	29	55	7	26	404	691	119	40	74	13	43

University of Texas classification: grade (I: superficial, not involving tendon, capsule, or bone, II: penetrating to tendon/capsule, III: penetrating to bone or joint).

ASO: arteriosclerosis obliterans; w/o: without

**Table:** Table 3-2 Pretreatment condition 2

a. Total																								
	Temperature ≥38°		Blood test								Hemodynamics								Infection*1)					
	(−)	(+)	WBC		CRP		Alb		Cr		ABI		TBI		SPP		Toe pressure		Local (foot)				Systemic	
			n	Median	n	Median	n	Median	n	Median	n	Median	n	Median	n	Median	n	Median	Uninfected	Skin or subcutaneous tissue (erythema)*2)		Deep tissue*3)	SIRS*4)	
																				≤2 cm	>2 cm		(+)	(−)
Rutherford 4	298	6	280	6235	274	0.5	261	3.7	274	1.225	123	0.56	12	0.425	91	21.0	12	56.0	–	–	–	–	3	300
Rutherford 5	782	25	780	6900	764	0.86	758	3.4	780	1.905	495	0.67	34	0.325	506	25.0	36	46.5	479	211	44	72	19	788
Rutherford 6	167	21	184	9005	183	3.56	178	3	181	1.27	100	0.68	8	0.25	112	22.0	8	23.0	47	45	38	58	26	162
Total	1247	52	1244	6930	1221	0.96	1197	3.4	1235	1.48	718	0.66	54	0.38	709	24.0	56	50.0	526	256	82	130	48	1250
b. ASO																								
	Temperature ≥38°		Blood test								Hemodynamics								Infection*1)					
	(−)	(+)	WBC		CRP		Alb		Cr		ABI		TBI		SPP		Toe pressure		Local (foot)				Systemic	
			n	Median	n	Median	n	Median	n	Median	n	Median	n	Median	n	Median	n	Median	Uninfected	Skin or subcutaneous tissue (erythema)*2)		Deep tissue*3)	SIRS*4)	
																				≤2 cm	>2 cm		(+)	(−)
Rutherford 4	294	6	276	6270	270	0.505	258	3.7	271	1.23	121	0.56	11	0.41	90	22.0	11	56.0	–	–	–	–	3	296
Rutherford 5	769	24	766	6900	750	0.855	744	3.4	766	1.965	483	0.67	34	0.325	496	26.0	36	46.5	474	205	44	69	16	777
Rutherford 6	166	21	183	9100	182	3.575	177	3	180	1.285	99	0.68	8	0.25	111	22.0	8	23.0	47	45	38	57	26	161
Total	1229	51	1225	6930	1202	0.97	1179	3.4	1217	1.5	703	0.65	53	0.375	697	24.0	55	48.5	521	250	82	126	45	1234

*1) Presence of infection was defined as by the presence of at least 2 of the following issues: ① Local swelling or induration, ② erythema >5 mm to ≤2 cm around the ulcer, ③ Local tenderness or pain, ④ Local warmth, ⑤ Purulent discharge (thick opaque to white, or sanguineous secretion)

*2) Local infection are skin and subcutaneous tissue was classified by the spreading of erythema (≤2 cm or >2 cm around the ulcer/gangrene.

*3) Local infection involving structures deeper than skin and subcutaneous tissues (e.g., abscess, osteomyelitis, septic arthritis, fasciitis).

*4) The signs of SIRS are manifested by two or more of the following: ① Temperature >38°C or <36°C, ② Heart rate >90 beats/min, ③ Respiratory rate >20 breaths/min or PaCO_2_<32 mmHg, ④ White blood cell count >12,000 or <4,000 cu/mm or 10% immature (band) forms.

ASO: arteriosclerosis obliterans; WBC: white blood cell; CRP: C reactive protein; Alb: albumin; Cr: creatinine; ABI: ankle brachial (pressure) index; TBI: toe brachial (pressure) index; SPP: skin perfusion pressure; SIRS: systemic inflammatory response syndrome

**Table:** Table 3-3 Pretreatment condition 3

a. Total																
	Diagnostic imaging			Site of occlusion			TASC II classification aortoiliac					TASC II classification femoropopliteal				
	IADSA	CTA	Others	Aortoiliac	Femoropop	Lower leg/foot	A	B	C	D	No lesion	A	B	C	D	No lesion
Rutherford 4	178	188	13	83	191	144	11	17	9	41	2	14	35	33	107	24
Rutherford 5	562	457	15	162	510	480	50	27	19	58	8	81	104	110	216	112
Rutherford 6	109	126	4	46	131	108	13	5	2	21	0	12	24	19	60	19
Total	849	771	32	291	832	732	74	49	30	120	10	107	163	162	383	155
b. ASO																
	Diagnostic imaging			Site of occlusion			TASC II classification aortoiliac					TASC II classification femoropopliteal				
	IADSA	CTA	Others	Aortoiliac	Femoropop	Lower leg/foot	A	B	C	D	No lesion	A	B	C	D	No lesion
Rutherford 4	177	185	13	83	189	142	11	17	9	41	2	14	34	33	106	22
Rutherford 5	552	451	15	161	506	470	50	27	19	57	8	80	104	109	213	106
Rutherford 6	108	126	4	46	131	107	13	5	2	21	0	12	24	19	60	18
Total	837	762	32	290	826	719	74	49	30	119	10	106	162	161	379	146

ASO: arteriosclerosis obliterans; IADSA: intra-arterial digital subtraction angiography, CTA: computed tomography angiography

**Table:** Table 3-4 Pretreatment condition 4

a. Total														
	Bollinger Score													
	Common femoral		Deep femoral		Superficial femoral: proximal		Superficial femoral: distal		Popliteal: proximal		Popliteal: distal		Tibioperoneal trunk	
	n	Median	n	Median	n	Median	n	Median	n	Median	n	Median	n	Median
Rutherford 4	70	2.0	70	1.0	71	3.0	72	6.0	70	4.0	69	2.0	65	2.0
Rutherford 5	383	1.0	381	1.0	383	3.0	383	4.0	383	3.0	388	2.0	379	3.0
Rutherford 6	89	1.0	89	1.0	89	3.0	89	5.0	89	3.0	88	2.0	87	3.0
Total	542	1.0	540	1.0	543	3.0	544	5.0	542	3.0	545	2.0	531	3.0
b. ASO														
	Bollinger Score													
	Common femoral		Deep femoral		Superficial femoral: proximal		Superficial femoral: distal		Popliteal: proximal		Popliteal: distal		Tibioperoneal trunk	
	n	Median	n	Median	n	Median	n	Median	n	Median	n	Median	n	Median
Rutherford 4	70	2.0	70	1.0	71	3.0	72	6.0	70	4.0	69	2.0	65	2.0
Rutherford 5	375	1.0	373	1.0	375	3.0	375	4.0	375	3.0	380	2.0	371	3.0
Rutherford 6	88	1.0	88	1.0	88	3.0	88	5.0	88	3.0	87	2.0	86	3.0
Total	533	1.0	531	1.0	534	3.0	535	5.0	533	3.0	536	2.0	522	3.0

ASO: arteriosclerosis obliterans

**Table:** Table 3-5 Pretreatment condition 5

a. Total														
	Bollinger Score													
	Posterior tibial: proximal		Posterior tibial: distal		Anterior tibial: proximal		Anterior tibial: distal		Peroneal: proximal		Peroneal: distal		Foot	
	n	Median	n	Median	n	Median	n	Median	n	Median	n	Median	n	Median
Rutherford 4	65	13.0	64	13.0	65	13.0	66	13.0	65	5.0	63	5.0	57	6.0
Rutherford 5	373	13.0	371	13.0	377	13.0	377	13.0	376	5.0	372	5.0	339	6.0
Rutherford 6	86	13.0	86	11.0	86	13.0	86	6.0	86	4.5	86	4.0	78	6.0
Total	524	13.0	521	13.0	528	13.0	529	13.0	527	5.0	521	5.0	474	6.0
b. ASO														
	Bollinger Score													
	Posterior tibial: proximal		Posterior tibial: distal		Anterior tibial: proximal		Anterior tibial: distal		Peroneal: proximal		Peroneal: distal		Foot	
	n	Median	n	Median	n	Median	n	Median	n	Median	n	Median	n	Median
Rutherford 4	65	13.0	64	13.0	65	13.0	66	13.0	65	5.0	63	5.0	57	6.0
Rutherford 5	365	13.0	363	13.0	369	13.0	369	13.0	368	5.0	364	5.0	332	6.0
Rutherford 6	85	13.0	85	9.0	85	13.0	85	6.0	85	5.0	85	4.0	77	6.0
Total	515	13.0	512	13.0	519	13.0	520	13.0	518	5.0	512	5.0	466	6.0

ASO: arteriosclerosis obliterans

**Table:** Table 3-6 SVS WIfI classification

a. Total																
	Wound				Ischemia				foot Infection				Stage			
	0	1	2	3	0	1	2	3	0	1	2	3	1	2	3	4
Rutherford 4	289	0	0	0	22	29	39	67	1	1	0	0	0	1	0	0
Rutherford 5	0	329	374	60	85	101	138	298	452	200	103	13	68	77	233	239
Rutherford 6	0	9	72	96	16	24	21	74	44	40	70	23	0	4	19	112
Total	289	338	446	156	123	154	198	439	497	241	173	36	68	82	252	351
b. ASO																
	Wound				Ischemia				foot Infection				Stage			
	0	1	2	3	0	1	2	3	0	1	2	3	1	2	3	4
Rutherford 4	285	0	0	0	22	28	39	66	1	1	0	0	0	1	0	0
Rutherford 5	0	325	368	56	83	98	138	290	447	195	102	10	66	77	230	231
Rutherford 6	0	9	71	96	16	24	21	73	44	40	69	23	0	4	19	111
Total	285	334	439	152	121	150	198	429	492	236	171	33	66	82	249	342

SVS: Society for Vascular Surgery; ASO: arteriosclerosis obliterans

Regarding the state of local tissue defect (Texas University Classification),^12)^ the most severe lesion, the main treatment target, was evaluated. SPP was measured on the foot (base of the toe, dorsum of the foot, or sole) and a lower value was adopted. To perform WIfI classification, the sites of ulcer and gangrene were registered separately. Although SPP is widely used as an objective index for evaluating ischemia in Japan, ischemic grading criteria using SPP are not shown in WIfI classification, in which TP is given top priority. Therefore, in the JCLIMB, the SPP value was converted to TP using the conversion equation SPP = 0.6853 × TP + 14.48 from the correlation data of SPP and TP reported in Japan,^13)^ and applied for WIfI ischemic grading (**Table 1-2-2**). Since the ischemic grade of WIfI classification based on the value of SPP was newly defined in the Japanese PAD guideline revised in 2022,^14)^ the classification method is changed in the annual report after 2020.

The lesion was considered infected when it showed two or more of the following findings: local swelling or induration, erythema >0.5cm around the ulcer, local tenderness or pain, local warmth, and purulent discharge (thick, opaque to white, or sanguineous secretion). In addition, local infections involving only the skin and the subcutaneous tissue, and those involving structures deeper than the skin and subcutaneous tissues, were registered separately. Local infections involving only the skin and the subcutaneous tissue were differentiated based on the size of the erythema around the ulcer, ≤2 or >2 cm. Systemic inflammatory response syndrome (SIRS), indicating systemic infection, was manifested by two or more of the following signs: temperature >38°C or <36°C, heart rate >90 beats/min, respiratory rate >20 breaths/min or PaCO_2_ <32 mmHg, and white blood cell count >12000 or <4000 cu/mm or 10% immature (band) forms. The arteries in the ankle joint region were classified as foot arteries.

In pretreatment, 62% (62%) of the patients were ambulatory, 22% (22%) were ambulatory/homebound, and 16% (16%) were non-ambulatory. On the Rutherford classification (R),^15)^ limbs with categories R4, R5, and R6 accounted for 23% (23%), 62% (62%), and 14% (15%) of the limbs, respectively. The median ABI, TBI, and SPP of the measured limbs were 0.66 (0.66), 0.38 (0.375), and 24 mmHg (24 mmHg), respectively. The occlusive legion was located in the aortoiliac artery in 22% (23%) of the limbs, in the femoropopliteal artery in 64% (64%) of the limbs, and in the crural or foot artery in 56% (56%) of the limbs. The multiple occlusive lesions were located in the aortoiliac artery and the femoropopliteal artery in 8% (8%) of limbs, in the aortoiliac artery and the crural or foot artery in 0.5% (0.5%), in the femoropopliteal artery and the crural or foot artery in 25% (26%), and in the aortoiliac artery and the femoropopliteal artery and the crural or foot artery in 5% (5%).

We were able to apply the WIfI classification with sufficient data to 753 limbs (739 limbs). On the WIfI classification, limbs with the stages 1, 2, 3, and 4 accounted for 9% (9%), 11% (11%), 33% (34%), and 47% (46%) of the limbs, respectively.

The problems and considerations on these spreadsheets are described below. In **Table 3-3**, the total number of limbs in TASC II classification differed compared to the number in each column of the site of occlusion. In the “aortoiliac” lesion, a decreased number of that in TASC II classification may have been due to input omission. In the “femoropopliteal” lesion, an increased number of that in TASC II may have been due to including the crural lesions. In **Table 3-6**, there was some dissociation between the R and Wound grades. This may be because of the R grade’s obscure definition. In **Table 3-6**, 123 limbs (121 limbs) were registered as ischemic grade 0 in WIfI classification. By definition, a limb with ischemic grade 0 has an ABI of 0.8 or higher or AP higher than 100 mmHg, if arterial calcification precludes reliable ABI or AP measurements, TP of 60 mmHg or higher or TcPO_2_ 60 mmHg or higher (SPP 50 mmHg or higher in the JCLIMB) (**Table 1-1-2**). There should be no limb with ischemic grade 0 since CLI registered in JCLIMB is defined according to TASC II. The limbs might be clinically judged to be CLI irrespective of the objective ischemic index, although details are unknown. In **Table 3-6**, there was 1 limb (1 limb), in which infection was confirmed in R4 limbs, despite the absence of a local wound by definition of R4. This may occur because tissue loss is not always requisite for fI grade. In **Table 3-6**, the numbers of wound, ischemia, foot Infection, and stage are different. This is because there were missing values in items required for grading.

### (3) Treatment

**Tables 4-1–4-6** show the CLI treatment data. Revascularizations of the affected limbs were performed in 97% (97%) of the registered limbs, and primary major amputations were performed in 2.0% (2.0%) of the registered limbs. Among the surgical reconstruction procedures, distal bypass accounted for 45% (44%). Endovascular treatment (EVT), including EVT alone and hybrid treatment with surgical reconstruction, accounted for 57% (57%) of the total revascularization procedures. EVT applied to the crural or foot artery accounted for 35% (35%) of the total EVT.

**Table:** Table 4 Treatment Table 4-1 Treatment 1

a. Total																				
	Treatment					Angiogenic therapy			Amputation							Reoperation				
	Pharmacological therapy	Angiogenic therapy	Arterial reconstruction	Major amputation	Lumber sympathectomy	Bone marrow	Peripheral blood	Others	Toe	Metatarsal	Chopart/Lisfranc	Syme	Below knee	Above knee or knee disarticulation	Hip disarticulation	Unknown	(−)	(+)		
																		1X	2X	3X≤
Rutherford 4	54	0	296	2	0	0	0	0	0	0	0	0	0	0	0	3	206	46	18	31
Rutherford 5	207	1	785	11	0	0	1	0	27	2	0	0	4	2	0	9	625	114	28	31
Rutherford 6	50	1	174	13	0	0	0	1	2	5	1	0	4	2	0	0	148	19	11	10
Total	311	2	1255	26	0	0	1	1	29	7	1	0	8	4	0	12	979	179	57	72
b. ASO																				
	Treatment					Angiogenic therapy			Amputation							Reoperation				
	Pharmacological therapy	Angiogenic therapy	Arterial reconstruction	Major amputation	Lumber sympathectomy	Bone marrow	Peripheral blood	Others	Toe	Metatarsal	Chopart/Lisfranc	Syme	Below knee	Above knee or knee disarticulation	Hip disarticulation	Unknown	(−)	(+)		
																		1X	2X	3X≤
Rutherford 4	54	0	292	2	0	0	0	0	0	0	0	0	0	0	0	2	204	46	18	30
Rutherford 5	202	1	773	11	0	0	1	0	27	2	0	0	4	2	0	9	614	111	28	31
Rutherford 6	49	1	173	13	0	0	0	1	2	5	1	0	4	2	0	0	147	19	11	10
Total	305	2	1238	26	0	0	1	1	29	7	1	0	8	4	0	11	965	176	57	71

ASO: arteriosclerosis obliterans

**Table:** Table 4-2 Treatment 2

a. Total															
	Bypass											TEA			EVT
	Aorta-aorta	Aorta (with suprarenal clamp)	Aorta-femoral*	Femora-proximal popliteal	Femoral-distal popliteal	Femoral-crural/foot	Popliteal-crural/foot	Anatomical others	Axillary-femoral	Femoral-femoral	Extraanatomical others	Aorta/iliac	Femoral/Popliteal	Others	
Rutherford 4	2	0	8	16	16	33	18	0	4	11	2	8	28	4	180
Rutherford 5	1	0	2	42	41	73	110	8	8	12	3	9	68	9	524
Rutherford 6	0	1	1	6	11	22	25	2	1	4	0	2	14	1	116
Total	3	1	11	64	68	128	153	10	13	27	5	19	110	14	820
b. ASO															
	Bypass											TEA			EVT
	Aorta-aorta	Aorta (with suprarenal clamp)	Aorta-femoral*	Femora-proximal popliteal	Femoral-distal popliteal	Femoral-crural/foot	Popliteal-crural/foot	Anatomical others	Axillary-femoral	Femoral-femoral	Extraanatomical others	Aorta/iliac	Femoral/Popliteal	Others	
Rutherford 4	2	0	7	15	16	32	17	0	4	11	2	8	28	4	179
Rutherford 5	1	0	2	42	40	71	107	8	8	12	3	9	68	9	516
Rutherford 6	0	1	1	6	11	22	25	2	1	4	0	2	14	1	115
Total	3	1	10	63	67	125	149	10	13	27	5	19	110	14	810

*Including aorta-femoral, aorta-iliac, ilio-femoral bypass.

ASO: arteriosclerosis obliterans; TEA: thromboendarterectomy; EVT: endovascular treatment/therapy

**Table:** Table 4-3 Treatment 3

a. Total																	
	EVT				Vascular material					Vein usage					Vein quality		
	Aorta/Iliac	Femoral/Popliteal	Tibioperoneal/Foot	Others	Polyester	ePTFE	Vein	Others	(−)	In-situ	Non-reversed	Reversed	Spliced	Patch	Patch	Good	Poor
Rutherford 4	60	84	69	11	13	36	79	10	42	12	24	23	3	16	11	61	5
Rutherford 5	125	298	250	5	11	65	257	13	52	30	111	68	17	29	21	212	17
Rutherford 6	38	63	50	0	3	8	67	7	22	11	31	18	2	7	5	56	4
Total	223	445	369	16	27	109	403	30	116	53	166	109	22	52	37	329	26
b. ASO																	
	EVT				Vascular material					Vein usage					Vein quality		
	Aorta/Iliac	Femoral/Popliteal	Tibioperoneal/Foot	Others	Polyester	ePTFE	Vein	Others	(−)	In-situ	Non-reversed	Reversed	Spliced	Patch	Patch	Good	Poor
Rutherford 4	60	84	69	10	13	35	77	10	42	12	24	21	3	16	11	59	5
Rutherford 5	125	297	244	3	11	65	250	13	51	30	107	66	17	28	20	206	17
Rutherford 6	38	63	49	0	3	8	67	7	22	11	31	18	2	7	5	56	4
Total	223	444	362	13	27	108	394	30	115	53	162	105	22	51	36	321	26

ASO: arteriosclerosis obliterans; EVT: endovascular treatment/therapy; ePTFE: expanded polytetrafluoroethylene

**Table:** Table 4-4 Treatment 4

a. Total																			
	Distal bypass																		
	Proximal anastomosis								Distal anastomosis		Distal anastomosis: site of crural artery				Distal anastomosis: site of foot artery				
	External iliac	Common femoral	Deep femoral	Superficial femoral	Proximal popliteal	Distal popliteal	Crural	Others	Crural	Foot	Tibioperoneal trunk	Posterior tibial	Anterior tibial	Peroneal	Posterior tibial	Anterior tibial	Peroneal	Dorsal pedis	Plantar
Rutherford 4	1	25	2	8	6	7	2	1	32	19	3	22	6	1	3	2	0	8	6
Rutherford 5	0	45	2	28	25	76	4	4	70	115	2	39	23	7	36	11	1	52	16
Rutherford 6	2	13	2	6	6	17	2	0	21	27	1	10	8	2	8	1	0	13	5
Total	3	83	6	42	37	100	8	5	123	161	6	71	37	10	47	14	1	73	27
b. ASO																			
	Distal bypass																		
	Proximal anastomosis								Distal anastomosis		Distal anastomosis: site of crural artery				Distal anastomosis: site of foot artery				
	External iliac	Common femoral	Deep femoral	Superficial femoral	Proximal popliteal	Distal popliteal	Crural	Others	Crural	Foot	Tibioperoneal trunk	Posterior tibial	Anterior tibial	Peroneal	Posterior tibial	Anterior tibial	Peroneal	Dorsal pedis	Plantar
Rutherford 4	1	25	2	8	6	6	1	1	30	19	3	21	5	1	3	2	0	8	6
Rutherford 5	0	43	2	28	25	73	4	4	67	113	2	39	20	7	36	11	1	51	15
Rutherford 6	2	13	2	6	6	17	2	0	21	27	1	10	8	2	8	1	0	13	5
Total	3	81	6	42	37	96	7	5	118	159	6	70	33	10	47	14	1	72	26

ASO: arteriosclerosis obliterans

**Table:** Table 4-5 Treatment 5

a. Total						
	Pharmacological therapy					
	Antiplatelet	Anticoagulant	Prostaglandin	Heparin	Statin	Others
Rutherford 4	73	8	7	3	16	14
Rutherford 5	281	20	28	18	46	35
Rutherford 6	71	2	5	4	13	3
Total	425	30	40	25	75	52
b. ASO						
	Pharmacological therapy					
	Antiplatelet	Anticoagulant	Prostaglandin	Heparin	Statin	Others
Rutherford 4	73	8	7	3	16	14
Rutherford 5	275	20	26	18	45	33
Rutherford 6	69	2	4	4	13	2
Total	417	30	37	25	74	49

ASO: arteriosclerosis obliterans

**Table:** Table 4-6 Treatment 6

a. Total				
	Femoro-proximal popliteal bypass	Femoro-distal popliteal bypass	Femoro-crural/foot bypass	Popliteal-crural/foot bypass
Polyester	0	1	0	1
ePTFE	50	12	3	5
Vein	16	60	125	148
Artery	1	0	0	4
Others	1	0	1	0
(−)	0	0	1	0
Total	68	73	130	158
b. ASO				
	Femoro-proximal popliteal bypass	Femoro-distal popliteal bypass	Femoro-crural/foot bypass	Popliteal-crural/foot bypass
Polyester	0	1	0	1
ePTFE	49	12	3	5
Vein	16	59	121	145
Artery	1	0	0	4
Others	1	0	1	0
(−)	0	0	1	0
Total	67	72	126	155

ePTFE: expanded polytetrafluoroethylene; ASO: arteriosclerosis obliterans

The problems and considerations on these spreadsheets are described below. In **Table 4-1**, the sum of the number of cells in treatment is larger than the number of registered limbs, 1299 (1280), because more than one treatment method can be selected. In **Table 4-1**, there was discrepancy in the number of major amputation to the number of detail of amputation. This is because major amputation can be selected in “treatment” linked to “surgery for chronic arterial occlusion” in the NCD, while detail of amputation is linked to “other vascular disease and related surgeries” in the NCD. In **Table 4-3**, how to use the autologous vein grafts is described when they were selected as vascular conduits. The sum of the number in the column of vein usage, “in-situ,” “nonreversed,” “reversed,” “spliced”, and “patch,” or the sum of vein quality is less than the sum of the number in the column of vein in vascular prosthesis. It is impossible because of selecting multiple vein usage for arterial reconstruction in a limb. It is assumed to be due to missing data. Vascular prosthesis (−) included an endarterectomy without a patch angioplasty. In **Table 4-4**, the sum of the number of proximal anastomosis is not equal to the sum of the number of distal anastomosis in R-4 or R-5. This was because multiple arteries could be selected in each anastomosis. The total number of distal anastomosis sites of foot artery is larger than that of distal anastomosis “foot.” This was because multiple sites were selected in dual bypass.

**Table 4-6** summarizes the vascular grafts used for the infrainguinal arterial reconstruction. Until 2019, when thromboendarterectomy (TEA) and bypass were performed at the same time, the materials for patch after TEA and bypass were entered without distinction. From 2020, the materials were entered separately for patch after TEA and bypass. However, this led to an increase in input errors. For example, the total number of vascular grafts in the column of femoral-proximal popliteal artery bypass was 64 (63) in **Table 4-2**, while the total number entered in graft materials of bypass was 47 (46) and the number entered only in patch materials with blank of bypass material section in patients undergoing bypass surgery was 21 (21). This is assumed due to input errors. Accordingly, in **Table 4-6**, the sum of the number of bypass materials in patients undergoing bypass surgery and the number of patch materials with blank of bypass material section in patients undergoing bypass surgery is shown. The input settings will be corrected after 2024, but a similar situation will occur until 2023. As multiple graft materials could be selected when multiple procedures were performed simultaneously, the number of graft materials for bypass is larger than the number of bypass surgeries.

### (4) Outcomes early (1 month) after treatment

**Tables 5-1–5-8** show the outcomes early (1 month) after treatment. At the time of summary count at the end of April 2022, follow-up data 1 month after treatment were obtained in 1063 limbs (82%), including 1045 limbs (82%) with ASO. Data were collected according to the severity of the local limb conditions (Rutherford classification) and treatment measures (EVT alone or surgical reconstruction with/without EVT). The mortality was 3.2% (3.2%) in the whole series, and 4.0% (4.1%) and 2.3% (2.1%) treated by EVT alone and by surgical reconstruction with/without EVT, respectively. The most common cause of death was cardiac disease, which accounted for 29% (30%) of all deaths. Postoperative complications were cardiac disease in 1.3% (1.2%), cerebrovascular disease in 1.6% (1.7%), pneumonia in 1.2% (1.1%), and wound complication in 4.8% (4.8%). Complications at the puncture site were noted in 0.7% (0.7%) of the limbs treated by EVT alone.

**Table:** Table 5 Outcomes early (1 month) treatment therapeutic measures: EVT (only EVT without surgical reconstruction) and surgical reconstruction (surgical reconstruction with or without EVT) Table 5-1 Life prognosis/causes of death

a. Total																
		Life prognosis			Causes of death											
		Alive	Dead	Intraoperative death	Cardiac disease	Cerebrovascular disease			Malignant neoplasm	Aortic aneurysm, dissection	Infection		Ischemic enteritis	Gastrointestinal bleeding	Others	Unknown
						Hemorrhage	Infarction	Unknown			Diseased limb	Others				
Local condition	Rutherford 4	200	7	0	1	0	0	0	0	0	1	1	0	0	3	1
	Rutherford 5	679	17	0	6	0	1	0	0	0	0	4	1	0	5	0
	Rutherford 6	150	10	0	3	0	0	0	0	0	0	3	1	1	2	0
Therapeutic measures	Non-reconstruction	33	1	0	0	0	0	0	0	0	0	1	0	0	0	0
	EVT	524	22	0	7	0	1	0	0	0	1	4	1	1	6	1
	Surgical reconstruction	472	11	0	3	0	0	0	0	0	0	3	1	0	4	0
	Total	1029	34	0	10	0	1	0	0	0	1	8	2	1	10	1
b. ASO																
		Life prognosis			Causes of death											
		Alive	Dead	Intraoperative death	Cardiac disease	Cerebrovascular disease			Malignant neoplasm	Aortic aneurysm, dissection	Infection		Ischemic enteritis	Gastrointestinal bleeding	Others	Unknown
						Hemorrhage	Infarction	Unknown			Diseased limb	Others				
Local condition	Rutherford 4	196	7	0	1	0	0	0	0	0	1	1	0	0	3	1
	Rutherford 5	667	16	0	6	0	1	0	0	0	0	3	1	0	5	0
	Rutherford 6	149	10	0	3	0	0	0	0	0	0	3	1	1	2	0
Therapeutic measures	Non-reconstruction	31	1	0	0	0	0	0	0	0	0	1	0	0	0	0
	EVT	517	22	0	7	0	1	0	0	0	1	4	1	1	6	1
	Surgical reconstruction	464	10	0	3	0	0	0	0	0	0	2	1	0	4	0
	Total	1012	33	0	10	0	1	0	0	0	1	7	2	1	10	1

EVT: endovascular treatment; ASO: arteriosclerosis obliterans

**Table:** Table 5-2 Perioperative complications 1

a. Total																
		Cardiac disease				Cerebrovascular disease				Pneumonia		Wound complication		Peripheral embolism		
		(−)	Angina	Serious arrhythmia	Myocardial infarction	(−)	TIA	Cerebral infarction		(−)	(+)	(−)	(+)	(−)	(+)	
								Functional loss (−)	Functional loss (+)						Minor (including blue toe)	Major
Local condition	Rutherford 4	199	2	0	0	198	1	0	2	199	2	190	11	198	1	2
	Rutherford 5	677	3	2	2	676	3	1	4	676	8	650	34	680	2	2
	Rutherford 6	152	0	3	0	149	0	1	5	153	2	150	5	153	0	2
Therapeutic measures	Non-reconstruction	11	0	0	0	11	0	0	0	11	0	11	0	11	0	0
	EVT	540	3	2	1	536	0	2	8	539	7	544	2	542	3	1
	Surgical reconstruction	477	2	3	1	476	4	0	3	478	5	435	48	478	0	5
	Total	1028	5	5	2	1023	4	2	11	1028	12	990	50	1031	3	6
b. ASO																
		Cardiac disease				Cerebrovascular disease				Pneumonia		Wound complication		Peripheral embolism		
		(−)	Angina	Serious arrhythmia	Myocardial infarction	(−)	TIA	Cerebral infarction		(−)	(+)	(−)	(+)	(−)	(+)	
								Functional loss (−)	Functional loss (+)						Minor (including blue toe)	Major
Local condition	Rutherford 4	195	2	0	0	194	1	0	2	195	2	186	11	194	1	2
	Rutherford 5	666	3	2	2	665	3	1	4	666	7	640	33	669	2	2
	Rutherford 6	151	0	3	0	148	0	1	5	152	2	149	5	152	0	2
Therapeutic measures	Non-reconstruction	11	0	0	0	11	0	0	0	11	0	11	0	11	0	0
	EVT	533	3	2	1	529	0	2	8	532	7	537	2	535	3	1
	Surgical reconstruction	468	2	3	1	467	4	0	3	470	4	427	47	469	0	5
	Total	1012	5	5	2	1007	4	2	11	1013	11	975	49	1015	3	6

TIA, transient ischemic attack; EVT, endovascular treatment; ASO: arteriosclerosis obliterans

**Table:** Table 5-3 Perioperative complications 2

a. Total															
		Hemorrhage			Site of bleeding			Outcome of bleeding				Complication due to contrast medium		Complication at puncture site	
		(−)	(+)	Unknown	Brain	GI tract	Others	Cured	Uncured	Dead	Others	(−)	(+)	(−)	(+)
Local condition	Rutherford 4	197	2	2	0	1	1	1	1	0	0	201	0	111	1
	Rutherford 5	673	9	2	0	3	6	8	1	0	0	682	2	452	3
	Rutherford 6	151	4	0	0	1	3	0	3	1	0	155	0	98	0
Therapeutic measures	Non-reconstruction	11	0	0	0	0	0	0	0	0	0	11	0	1	0
	EVT	538	6	2	0	3	3	2	3	1	0	546	0	542	4
	Surgical reconstruction	472	9	2	0	2	7	7	2	0	0	481	2	118	0
	Total	1021	15	4	0	5	10	9	5	1	0	1038	2	661	4
b. ASO															
		Hemorrhage			Site of bleeding			Outcome of bleeding				Complication due to contrast medium		Complication at puncture site	
		(−)	(+)	Unknown	Brain	GI tract	Others	Cured	Uncured	Dead	Others	(−)	(+)	(−)	(+)
Local condition	Rutherford 4	193	2	2	0	1	1	1	1	0	0	197	0	110	1
	Rutherford 5	662	9	2	0	3	6	8	1	0	0	671	2	446	3
	Rutherford 6	150	4	0	0	1	3	0	3	1	0	154	0	97	0
Therapeutic measures	Non-reconstruction	11	0	0	0	0	0	0	0	0	0	11	0	1	0
	EVT	531	6	2	0	3	3	2	3	1	0	539	0	535	4
	Surgical reconstruction	463	9	2	0	2	7	7	2	0	0	472	2	117	0
	Total	1005	15	4	0	5	10	9	5	1	0	1022	2	653	4

GI: gastrointestinal; EVT: endovascular treatment; ASO: arteriosclerosis obliterans

**Table:** Table 5-4 Hemodynamics

a. Total													
		Immediate after the treatment						1 month after the treatment					
		ABI		Ankle pressure		SPP		ABI		Ankle pressure		SPP	
		n	Median	n	Median	n	Median	n	Median	n	Median	n	Median
Local condition	Rutherford 4	103	0.82	91	107.0	45	40.0	66	0.88	62	121.0	26	40.0
	Rutherford 5	364	0.87	317	119.0	258	42.0	240	0.91	204	121.0	159	46.0
	Rutherford 6	57	0.85	50	112.5	55	38.0	26	0.87	21	113.0	19	41.0
Therapeutic measures	Non-reconstruction	13	0.81	11	127.0	9	45.0	7	0.89	6	128.0	5	26.0
	EVT	298	0.86	255	118.0	179	39.0	190	0.91	161	119.0	100	48.0
	Surgical reconstruction	213	0.88	192	115.0	170	44.0	135	0.90	120	123.0	99	43.0
	Total	524	0.86	458	117.5	358	41.0	332	0.90	287	120.0	204	45.0
b. ASO													
		Immediate after the treatment						1 month after the treatment					
		ABI		Ankle pressure		SPP		ABI		Ankle pressure		SPP	
		n	Median	n	Median	n	Median	n	Median	n	Median	n	Median
Local condition	Rutherford 4	102	0.82	90	107.0	44	40.0	66	0.88	62	121.0	26	40.0
	Rutherford 5	359	0.87	312	118.5	254	42.0	237	0.91	201	121.0	156	46.0
	Rutherford 6	57	0.85	49	112.0	54	38.0	26	0.87	21	113.0	19	41.0
Therapeutic measures	Non-reconstruction	12	0.80	10	120.5	8	46.0	6	0.95	5	131.0	4	32.0
	EVT	294	0.85	250	117.5	178	39.0	188	0.90	159	119.0	99	48.0
	Surgical reconstruction	212	0.88	191	115.0	166	44.0	135	0.90	120	123.0	98	43.0
	Total	518	0.86	451	117.0	352	41.0	329	0.90	284	120.0	201	45.0

ABI: ankle brachial (pressure) index; SPP: skin perfusion pressure; EVT: endovascular treatment; ASO: arteriosclerosis obliterans

**Table:** Table 5-5 Condition of the limb

a. Total																		
		Bypass graft/EVT condition							Clinical symptom of the limb			Ischemic wound				Ambulatory function at discharge (Taylor's classification)		
		Good	Stenosis	Occlusion	Deterioration	Anastomosis disruption (aneurysm)	Infection	Others	Improved	No change	Deteriorated	Cured	Uncured		Unknown	Ambulatory	Ambulatory/ homebound	Nonambulatory
													Improved	Deteriorated				
Local condition	Rutherford 4	187	3	4	0	0	2	4	182	12	10	132	45	24	3	144	32	31
	Rutherford 5	627	19	22	1	2	3	11	577	94	18	173	377	133	6	412	143	141
	Rutherford 6	130	7	7	0	1	1	3	111	29	10	12	83	54	1	53	46	61
Therapeutic measures	Non-reconstruction	0	0	0	0	0	0	0	18	3	1	5	13	4	0	16	5	13
	EVT	506	16	15	1	0	0	10	431	88	24	149	247	140	7	294	101	151
	Surgical reconstruction	438	13	18	0	3	6	8	421	44	13	163	245	67	3	299	115	69
	Total	944	29	33	1	3	6	18	870	135	38	317	505	211	10	609	221	233
b. ASO																		
		Bypass graft/EVT condition							Clinical symptom of the limb			Ischemic wound				Ambulatory function at discharge (Taylor's classification)		
		Good	Stenosis	Occlusion	Deterioration	Anastomosis disruption (aneurysm)	Infection	Others	Improved	No change	Deteriorated	Cured	Uncured		Unknown	Ambulatory	Ambulatory/ homebound	Nonambulatory
													Improved	Deteriorated				
Local condition	Rutherford 4	183	3	4	0	0	2	4	178	12	10	129	44	24	3	141	31	31
	Rutherford 5	619	18	21	1	2	3	10	569	90	17	171	370	129	6	406	140	137
	Rutherford 6	129	7	7	0	1	1	3	110	29	10	12	82	54	1	52	46	61
Therapeutic measures	Non-reconstruction	0	0	0	0	0	0	0	17	2	1	4	13	3	0	16	4	12
	EVT	500	15	15	1	0	0	10	426	86	24	149	241	139	7	290	100	149
	Surgical reconstruction	431	13	17	0	3	6	7	414	43	12	159	242	65	3	293	113	68
	Total	931	28	32	1	3	6	17	857	131	37	312	496	207	10	599	217	229

EVT, endovascular treatment; ASO: arteriosclerosis obliterans

**Table:** Table 5-6 Revision of treatment

a. Total																		
		Revision for those excluding good bypass graft/EVT condition		Minor reintervention (revision for stenosis)				Major reintervention (revision for occlusion)								Major amputation		
		(+)	(−)	(−)	Patch plasty	EVT	Others	(−)	Thrombectomy (±patch plasty)	Thrombolysis	EVT	Re-bypass	Jump bypass	Interposition	Others	(−)	(+)	
																	Due to preoperative sound	Due to new wound
Local condition	Rutherford 4	5	7	197	0	2	0	191	2	0	2	2	0	1	1	196	6	3
	Rutherford 5	31	24	654	2	21	0	647	4	1	8	10	4	1	2	665	20	4
	Rutherford 6	11	7	136	0	8	1	137	0	0	0	3	1	1	3	136	13	1
Therapeutic measures	Non-reconstruction	0	0	0	0	0	0	0	0	0	0	0	0	0	0	23	0	0
	EVT	20	20	521	0	22	0	516	0	1	7	11	3	0	5	509	30	4
	Surgical reconstruction	27	18	466	2	9	1	459	6	0	3	4	2	3	1	465	9	4
	Total	47	38	987	2	31	1	975	6	1	10	15	5	3	6	997	39	8
b. ASO																		
		Revision for those excluding good bypass graft/EVT condition		Minor reintervention (revision for stenosis)				Major reintervention (revision for occlusion)								Major amputation		
		(+)	(−)	(−)	Patch plasty	EVT	Others	(−)	Thrombectomy (±patch plasty)	Thrombolysis	EVT	Re-bypass	Jump bypass	Interposition	Others	(−)	(+)	
																	Due to preoperative sound	Due to new wound
Local condition	Rutherford 4	5	7	193	0	2	0	187	2	0	2	2	0	1	1	192	6	3
	Rutherford 5	31	21	644	2	20	0	637	4	1	7	10	4	1	2	652	20	4
	Rutherford 6	11	7	135	0	8	1	136	0	0	0	3	1	1	3	135	13	1
Therapeutic measures	Non-reconstruction	0	0	0	0	0	0	0	0	0	0	0	0	0	0	21	0	0
	EVT	20	19	515	0	21	0	509	0	1	7	11	3	0	5	502	30	4
	Surgical reconstruction	27	16	457	2	9	1	451	6	0	2	4	2	3	1	456	9	4
	Total	47	35	972	2	30	1	960	6	1	9	15	5	3	6	979	39	8

EVT: endovascular treatment; ASO: arteriosclerosis obliterans

**Table:** Table 5-7 Condition of contralateral limb

a. Total																		
		Contralateral limb occlusive lesions							Treatment for contralateral limb									
		(−)	(+)						Unnecessary	(+)								
			Asymptomatic	Intermittent claudication	CLI			Post treatment		Pharmacological therapy	Angiogenic therapy	EVT	Surgical bypass	Minor amputation	Major amputation	Lumbar sympathectomy	Necessary but no treatment	Others
					R4	R5	R6											
Local condition	Rutherford 4	87	52	9	4	6	1	27	11	70	0	18	14	3	3	0	1	0
	Rutherford 5	234	219	23	8	42	2	138	71	265	2	92	53	22	19	0	12	2
	Rutherford 6	47	58	4	2	8	4	27	10	71	0	22	13	3	9	0	2	1
Therapeutic measures	Non-reconstruction	15	9	0	0	1	0	5	0	10	1	1	0	1	2	0	0	0
	EVT	177	181	14	8	29	4	107	50	222	1	91	26	19	19	0	8	1
	Surgical reconstruction	176	139	22	6	26	3	80	42	174	0	40	54	8	10	0	7	2
	Total	368	329	36	14	56	7	192	92	406	2	132	80	28	31	0	15	3
b. ASO																		
		Contralateral limb occlusive lesions							Treatment for contralateral limb									
		(−)	(+)						Unnecessary	(+)								
			Asymptomatic	Intermittent claudication	CLI			Post treatment		Pharmacological therapy	Angiogenic therapy	EVT	Surgical bypass	Minor amputation	Major amputation	Lumbar sympathectomy	Necessary but no treatment	Others
					R4	R5	R6											
Local condition	Rutherford 4	83	52	9	4	6	1	27	11	70	0	18	14	3	3	0	1	0
	Rutherford 5	231	212	23	8	40	2	137	70	257	2	91	52	22	19	0	12	2
	Rutherford 6	47	58	4	2	8	4	26	10	70	0	21	13	3	9	0	2	1
Therapeutic measures	Non-reconstruction	14	8	0	0	1	0	5	0	9	1	1	0	1	2	0	0	0
	EVT	175	179	14	8	28	4	105	50	217	1	89	26	19	19	0	8	1
	Surgical reconstruction	172	135	22	6	25	3	80	41	171	0	40	53	8	10	0	7	2
	Total	361	322	36	14	54	7	190	91	397	2	130	79	28	31	0	15	3

CLI: critical limb ischemia; EVT: endovascular treatment; ASO: arteriosclerosis obliterans

**Table:** Table 5-8 Malignant neoplasm

a. Total															
		Newly diagnosed malignant neoplasm			Sites of newly diagnosed malignant neoplasm										
		(−)	(+)	Unknown	Head and neck	Esophagus	Lung	Stomach	Hepatobiliary pancreas	Colon	Breast	Uterus	Ovarium	Prostate	Others
Local condition	Rutherford 4	205	1	1	0	0	1	0	0	0	0	0	0	0	0
	Rutherford 5	692	2	2	0	0	2	0	0	0	0	0	0	0	0
	Rutherford 6	160	0	0	0	0	0	0	0	0	0	0	0	0	0
Therapeutic measures	Non-reconstruction	34	0	0	0	0	0	0	0	0	0	0	0	0	0
	EVT	544	1	1	0	0	1	0	0	0	0	0	0	0	0
	Surgical reconstruction	479	2	2	0	0	2	0	0	0	0	0	0	0	0
	Total	1057	3	3	0	0	3	0	0	0	0	0	0	0	0
b. ASO															
		Newly diagnosed malignant neoplasm			Sites of newly diagnosed malignant neoplasm										
		(−)	(+)	Unknown	Head and neck	Esophagus	Lung	Stomach	Hepatobiliary pancreas	Colon	Breast	Uterus	Ovarium	Prostate	Others
Local condition	Rutherford 4	201	1	1	0	0	1	0	0	0	0	0	0	0	0
	Rutherford 5	679	2	2	0	0	2	0	0	0	0	0	0	0	0
	Rutherford 6	159	0	0	0	0	0	0	0	0	0	0	0	0	0
Therapeutic measures	Non-reconstruction	32	0	0	0	0	0	0	0	0	0	0	0	0	0
	EVT	537	1	1	0	0	1	0	0	0	0	0	0	0	0
	Surgical reconstruction	470	2	2	0	0	2	0	0	0	0	0	0	0	0
	Total	1039	3	3	0	0	3	0	0	0	0	0	0	0	0

EVT: endovascular treatment; ASO: arteriosclerosis obliterans

The median ABI and SPP of the measured limbs, immediately after treatment and 1 month after treatment, were 0.86 (0.86) and 0.90 (0.90) and 41 mmHg (41 mmHg) and 45 mmHg (45 mmHg), respectively. Stenosis, occlusion, infection, or other trouble occurred after revascularization by EVT alone in 7.7% (7.6%) and by surgical reconstruction with/without EVT in 9.9% (9.6%). Secondary major amputation rate was 6.3% (6.3%) in EVT alone and 2.7% (2.7%) in surgical reconstruction with/without EVT. When ambulatory function at discharge was compared to that before surgery, the rate of ambulatory patients changed from 62% (62%) to 57% (57%), ambulatory/homebound patients from 22% (22%) to 21% (21%), and nonambulatory patients from 16% (16%) to 22% (22%).

The problems, comments, and considerations on these spreadsheets are described below. The number of “bypass graft/EVT condition,” “clinical limb symptoms,” “ischemic wound,” and “ambulatory function at discharge” did not match (**Table 5-5**). The total number of “ambulatory function at discharge” was 1063 (1045), which was equal to the number of life prognoses (**Table 5-1**), indicating no “unused.” The number of “bypass graft/EVT condition” was not equal to the number of “ambulatory function at discharge” because the objectives of “bypass graft/EVT condition” were limbs of survivors with arterial reconstruction and because more than one condition could be selected. The numbers of “clinical symptoms of limb” and “ischemic wound” were not identical. They must be identical because their objective was survivor without major amputations. This is speculated to be due to the presence of “unused” in dead cases before registration. The number of limbs of survivors with EVT was 524 (517 limbs) (**Table 5-1**), which was 19 (19) limbs less than the sum of the number in the column of minor reintervention or major reintervention in the row of limbs with EVT, 543 limbs (536 limbs) (**Table 5-6**). The number of limbs of survivors with surgical reconstruction was 472 (464 limbs) (**Table 5-1**), which was 6 (6) limbs less than the sum of the number in the column of minor reintervention or major reintervention in the row of limbs with surgical reconstruction, 478 limbs (469 limbs) (**Table 5-6**). This is speculated to be due to death after reintervention. In **Table 5-6**, the objective for input of “revision for those excluding good bypass graft/EVT condition” is limb registered in stenosis, occlusion, deterioration, anastomosis disruption (aneurysm), infection, and others of “bypass graft/EVT condition”. The number of “bypass graft/EVT condition” of surgical reconstruction in **Table 5-5** does not match the number of “revision for those excluding good bypass graft/EVT condition” in **Table 5-6**. This is because multiple items can be selected in “bypass graft/EVT condition.” The total number of “the Contralateral limb occlusive lesions” in **Table 5-7** is 61 limbs (61 limbs) less than “life prognosis” in **Table 5-1**. When the bilateral limbs underwent revascularization simultaneously, all cases were eligible until 2019, but in 2020, it has not be set for “the Contralateral limb occlusive lesions” in the JCLIMB not to be expanded in cases of bilateral CLI. The sum of the number of “treatment for contralateral limb” is less than that of “the Contralateral limb occlusive lesions” because the objectives of “treatment for contralateral limb” excluded the limbs with no objective lesion in “the Contralateral limb occlusive lesions”. Because multiple registrations were possible, the sum of the number of “treatment for contralateral limb” was more than that of the limbs with occlusive lesions in the contralateral limb. The input settings will be revised in 2024. When a patient died within 1 month, the information of “newly diagnosed malignant neoplasm” at death was registered in **Table 5-8**.

In addition to the above, there were some parts where the total number does not match in **Tables 5-1**–**5-8**. It might be because several items had multiple choices or missing values.

## 4. Conclusions

Vascular surgeons’ contribution in participating facilities registered a sufficient amount of detailed data during busy clinical practice, which has been gradually clarifying the current status of CLI treatment in Japan. Data on CLI in 2020 were clarified, after annual data in 2013–2019. The JCLIMB Committee is planning to continue publishing an annual report in the future. In 2017, the new concept, “chronic limb threatening ischemia,” was proposed instead of CLI^16)^ and a new clinical guideline, the Global Vascular Guideline, was published instead of TASC in 2019.^17)^ The full name of the JCLIMB has been changed to “Japan Chronic Limb Threatening Ischemia Database” and the data format has been revised to register the survey items according to the Global Vascular Guideline, which can be used in 2021.

The JCLIMB Committee expects that these study results will be fed back to clinical situations to help develop medical care for CLI, and several studies using the data of JCLIMB are under way. The papers regarding 30 days’ and 2 years’ prediction model were published^18^^,^^19)^ and an app of the JCLIMB risk calculator based on the papers was developed. Anybody can use it now. Finally, a part of the purpose of the JCLIMB has been returned to the doctors working in the real clinical field. We hope that this project will contribute to the development of CLI treatment in Japan.

- “CLI” was used in this paper because the objectives registered in 2020 were based on CLI defined by TASC II.
- In this aggregation, it was found that some of the data were unreliable due to a setting error in the input method. We are extremely responsible for the loss of valuable information, and we deeply regret it.

## 5. Participant Facilities (85 facilities in the order of the Japanese syllabary by prefecture, corporate names are omitted as a rule)

Department of Cardiovascular Surgery, Sapporo Teishinkai Hospital

Department of Vascular Surgery, Asahikawa Medical University Hospital

Department of Cardiovascular Surgery, National Hospital Organization Obihiro Hospital

Department of Cardiovascular Surgery, Nayoro City General Hospital

Department of Cardiovascular Surgery, Hirosaki University Hospital

Department of Surgery, Iwate Prefectural Isawa Hospital

Department of Surgery, Iwate Prefectural Chubu Hospital

Department of Cardiovascular Surgery, Sendai City Hospital

Department of Transplantation, Reconstruction and Endoscopic Surgery, Tohoku University Hospital

Department of Cardiovascular Surgery, Saiseikai Yamagata Saisei Hospital

Department of Vascular and Endovascular Surgery, Ibaraki Prefectural Central Hospital

Department of Cardiac and Vascular Surgery, Dokkyo Medical University Hospital

Department of Vascular and Endovascular Surgery, International University of Health and Welfare Narita Hospital

Department of Vascular Surgery, Saiseikai Kawaguchi General Hospital

Department of Vascular Surgery, Saitama Medical Center, Saitama Medical University

Department of Cardiovascular Surgery, Saitama Medical Center, Jichi Medical University

Department of Cardiovascular Surgery, Jichi Medical University

Department of Surgery, Saitama City Hospital

Department of Cardiovascular Surgery, Shimada General Hospital

Department of Cardiovascular Surgery, Chiba Cerebral and Cardiovascular Center

Department of Cardiovascular Surgery, Itabashi Chuo Medical Center

Department of Cardiovascular Surgery, IMS Tokyo Katsushika General Hospital

Department of Vascular Surgery, Edogawa Hospital

Department of Surgery, Tokyo Metropolitan Health and Medical Treatment Corporation, Okubo Hospital

Department of Cardiovascular Surgery, Kyorin University

Department of Surgery, Keio University School of Medicine

Department of Vascular Surgery, International University of Health and Welfare, Mita Hospital

Department of Vascular Surgery, Tokyo Medical and Dental University

Department of Cardiovascular Surgery, Tokyo Medical University Hachioji Medical Center

Department of Cardiovascular Surgery, Tokyo Medical University Hospital

Department of Vascular Surgery, The Jikei University Kashiwa Hospital

Department of Vascular Surgery, The Jikei University Hospital

Department of Vascular Surgery, The University of Tokyo Hospital

Department of Cardiovascular Surgery, Tokyo Rinkai Hospital

Department of Vascular Surgery, Nihon University Itabashi Hospital

Department of Surgery, Shonan Kamakura General Hospital

Department of Cardiovascular Surgery, St. Marianna University School of Medicine

Department of Surgery, Tomei Atsugi Hospital

Department of Cardiovascular Surgery, Yokosuka General Hospital Uwamachi

Department of Cardiovascular Surgery, National Hospital Organization, Kanazawa Medical Center

Department of Cardiovascular Surgery, Kanazawa University Hospital

Department of Cardiovascular Surgery, Shizuoka Red Cross Hospital

Department of Vascular Surgery, Aichi Medical University Hospital

Department of Vascular Surgery, Ichinomiya Municipal Hospital

Department of Vascular Surgery, Japanese Red Cross Nagoya Daiichi Hospital

Department of Vascular Surgery, Nagoya University Hospital

Department of Vascular Surgery, Osaka Rosai Hospital

Department of Vascular Surgery, Aijinkai Inoue Hospital

Department of Vascular Surgery, Nippon Life Hospital

Department of Vascular Surgery, Kansai Medical University Medical Center

Department of Cardiovascular Surgery, Toyonaka Municipal Hospital

Department of Cardiovascular Surgery, Suita Tokushukai Hospital

Department of Cardiovascular Surgery, Tsukazaki Hospital

Department of Cardiovascular Surgery, Higashiosaka City Medical Center

Department of Cardiovascular Surgery, Kobe University Hospital

Department of Cardiovascular Surgery, Nara Prefecture Western Medical Center

Department of Thoracic and Cardiovascular Surgery, Wakayama Medical University Hospital

Department of Cardiovascular Surgery, Tottori Prefectural Kousei Hospital

Department of Cardiovascular Surgery, Tottori Prefectural Central Hospital

Department of Cardiovascular Surgery, Okayama University Hospital

Department of Cardiovascular Surgery, Kawasaki Medical School General Medical Center

Department of Cardiovascular Surgery, Kawasaki Medical School Hospital

Department of Cardiovascular and Respiratory Surgery, Hiroshima Prefectural Hospital

Department of Cardiovascular Surgery, National Hospital Organization, Higashihiroshima Medical Center

Department of Cardiovascular Surgery, Hiroshima University Hospital

Department of Surgery, Saiseikai Yamaguchi General Hospital

Department of Surgery, Yamaguchi University Hospital

Department of Cardiovascular Surgery, Ehime Prefectural Central Hospital

Department of Cardiovascular Surgery, Matsuyama Shimin Hospital

Department of Vascular Surgery, Matsuyama Red Cross Hospital

Department of Cardiovascular Surgery, Kochi Health Sciences Center

Department of Cardiovascular Surgery, Kochi University Hospital

Department of Vascular Surgery, National Hospital Organization, Kyushu Medical Center

Department of Surgery and Science, Kyushu University Hospital

Department of Cardiovascular Surgery, Kurume University Hospital

Department of Vascular Surgery, Kokura Memorial Hospital

Department of Surgery, Saiseikai Fukuoka General Hospital

Department of Surgery, Saiseikai Yahata General Hospital

Department of Vascular Surgery, Fukuoka City Hospital

Department of Vascular Surgery, National Hospital Organization, Fukuoka Higashi Medical Center

Department of Cardiovascular Surgery, Saga-Ken Medical Center Koseikan

Department of Surgery, Saiseikai Karatsu Hospital

Department of Cardiovascular Surgery, Sasebo Chuo Hospital

Department of Vascular Surgery, Kumamoto Rehabilitation Hospital

Department of Cardiovascular Surgery, Oita Oka Hospital

## 6. JCLIMB Committee, NCD JCLIMB Analytical Team

### (1) JCLIMB Committee

Shinsuke Mii (Chairman), Akio Kodama (Vice Chairman), Atsuhisa Ishida, Yoshinori Inoue, Hisashi Uchida, Takao Ohki, Sosei Kuma, Koji Kurosawa, Michinari Kono, Hiroyoshi Komai, Shinsuke Kikuchi, Taku Kokubo, Takashi Shibuya, Shunya Shindo, Ikuo Sugimoto, Juno Deguchi, Katsuyuki Hoshina, Hirofumi Midorikawa, Terutoshi Yamaoka, Hiroya Yamashita, Yasuhiro Yunoki, and Tetsuro Miyata (Observer)

### (2) NCD JCLIMB Analytical Team

Arata Takahashi and Hiroaki Miyata

## Disclosure Statement

The authors have no conflict of interest.

## Additional Remarks

This report was authorized by the institutional review board of Saiseikai Yahata General Hospital (Authorization No. 201).
